# Supplementary material for: α-hemolysin polymorphisms in methicillin-resistant Staphylococcus aureus clinical isolates regulate ADAM10-dependent neutrophil IL-1β secretion
Source: bioRxiv. 2025 Dec 14:2025.12.13.694139. Preprint. [Version 1] doi: 10.64898/2025.12.13.694139 (PMC12710830; doi:10.64898/2025.12.13.694139)
Supplement: Supplement 1 — Figure S1. USA300 keratitis phenotype is reproducible with 8325–4 keratitis and with topical infection. A-D. A 2 μL inoculum of 5×10^3^ CFU laboratory S. aureus strain 8325–4 was injected into the corneal stroma C57BL/6 or IL-1a/b−/− mice and examined after 24 h. A. Representative images of infected corneas. B. Quantification of viable bacteria in infected corneas by CFU. C. Flow cytometry quantification of neutrophils and monocytes in infected corneas. D. Whole eyes were enucleated, sectioned, and stained histological examination. Representative images are shown of H&E- and crystal violet-staining as well as immunohistochemistry using anti-Ly6G to show infiltrating neutrophils. E. A 5 μL inoculum of 1×10^6^ CFU 8325–4 strain was applied to the surface of abraded corneas from C57BL/6, Myd88−/−, or IL1R1−/− mice and examined after 24 h by immunohistochemistry. Representative images are shown of H&E staining. Figure S2. Flow Cytometry gating strategy. Representative flow cytometry gating strategy for identification of IL-1β+ neutrophils and monocytes in infected corneas. Total cells from infected corneas were gated on single, live cells and gated for total CD45+ myeloid cells, Ly6G+/CD11b+ neutrophils and Ly6C+/CD11b+ total monocytes. Figure S3. Rabbit RBC lysis, Inflammasome inhibition and neutrophil elastase and myeloperoxidase (MPO) production A. Conditioned media from USA300, ΔHla, and MRSA clinical isolates were serially diluted prior to incubation with RBCs for 1 h. Intact RBCs were centrifuged and release hemoglobin was assessed by reading optical density. Results are normalized to RBCs treated with triton-x. B-D. LPS-primed BMNs from C57BL/6 were incubated with stimulated for 1 h with 3 mM ATP or 1:10 diluted conditioned media from USA300, ΔHla, and MRSA clinical isolates in the presence of 2 μM NLRP3 inhibitor MCC950, 5 μM caspase-1 inhibitor YVAD, or 10 μM GSDMD inhibitor Disulfiram. Neutrophil Elastase, MPO, and IL-1β in culture supernatatants were quantified b [file media-1.pdf]

Figure S1

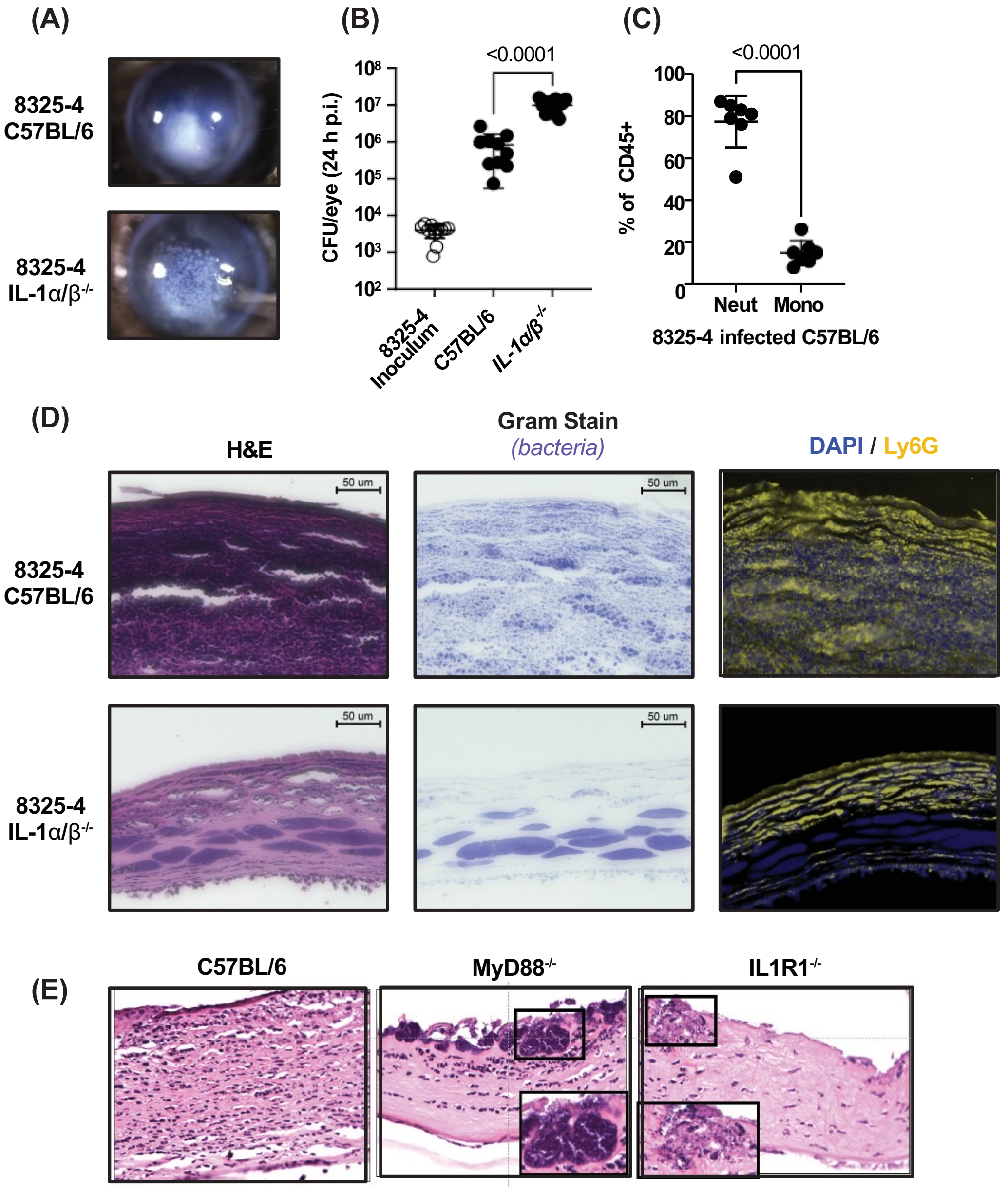

(A)
8325-4 C57BL/6
8325-4 IL-1α/β-/-
(B)
<0.0001
CFU/eye (24 h p.i.)
10^8
10^7
10^6
10^5
10^4
10^3
10^2
8325-4 Inoculum
C57BL/6
IL-1α/β-/-
(C)
<0.0001
% of CD45+
100
80
60
40
20
0
Neut
Mono
8325-4 infected C57BL/6
(D)
H&E
Gram Stain
(bacteria)
DAPI / Ly6G
8325-4 C57BL/6
8325-4 IL-1α/β-/-
50 um
(E)
C57BL/6
MyD88-/-
IL1R1-/-

Figure S2

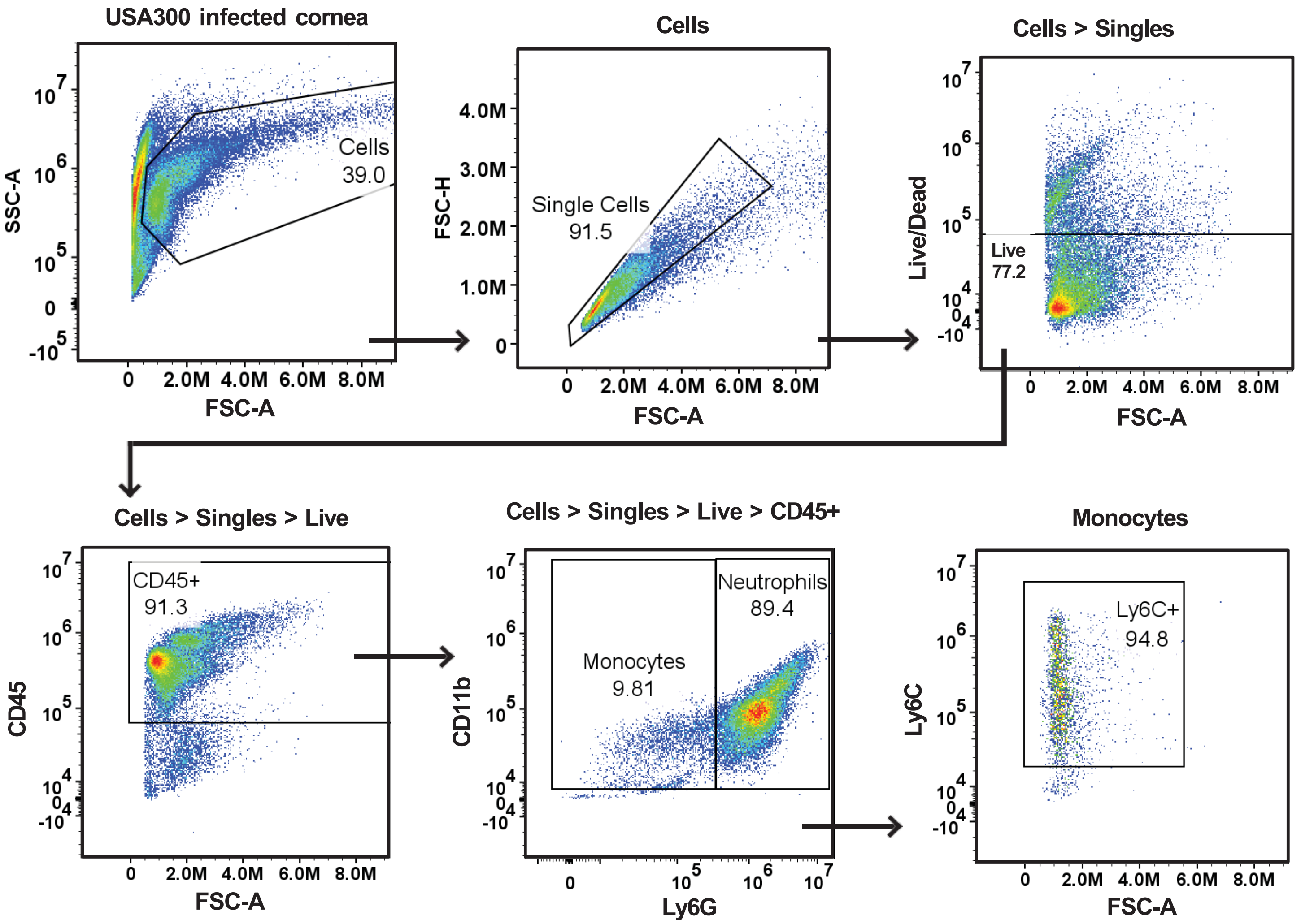

USA300 infected cornea
Cells
39.0
SSC-A
FSC-A
Cells
Single Cells
91.5
FSC-H
FSC-A
Cells > Singles
Live
77.2
Live/Dead
FSC-A
Cells > Singles > Live
CD45+
91.3
CD45
FSC-A
Cells > Singles > Live > CD45+
Monocytes
9.81
Neutrophils
89.4
CD11b
Ly6G
Monocytes
Ly6C+
94.8
Ly6C
FSC-A

Figure S3

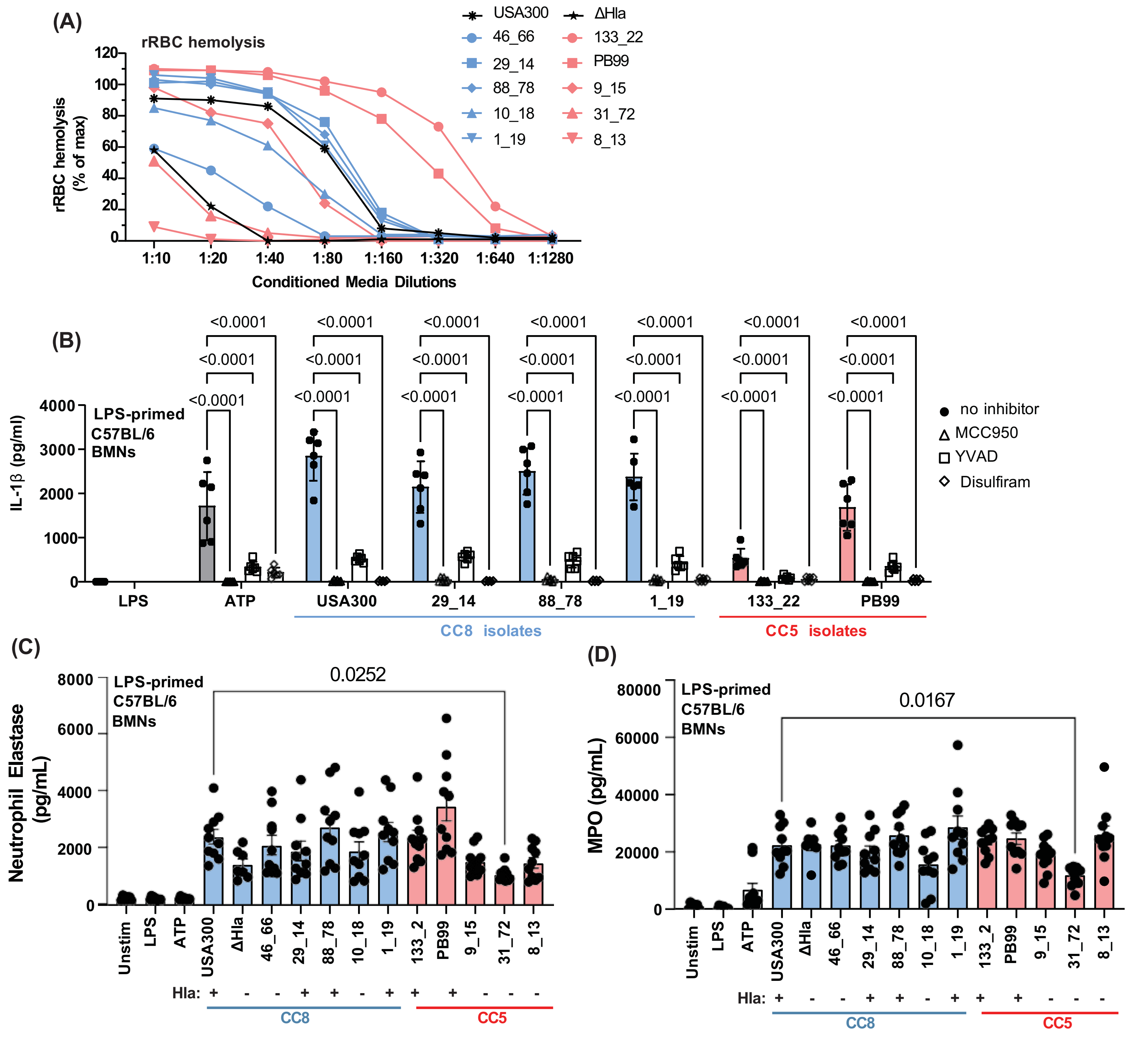

(A)
rRBC hemolysis
rRBC hemolysis (% of max)
120
100
80
60
40
20
0
1:10
1:20
1:40
1:80
1:160
1:320
1:640
1:1280
Conditioned Media Dilutions
USA300
ΔHla
46_66
133_22
29_14
PB99
88_78
9_15
10_18
31_72
1_19
8_13
(B)
IL-1β (pg/ml)
4000
3000
2000
1000
0
LPS-primed C57BL/6 BMNs
<0.0001
LPS
ATP
USA300
29_14
88_78
1_19
133_22
PB99
CC8 isolates
CC5 isolates
no inhibitor
MCC950
YVAD
Disulfiram
(C)
Neutrophil Elastase (pg/mL)
8000
6000
4000
2000
0
LPS-primed C57BL/6 BMNs
0.0252
Unstim
LPS
ATP
USA300
ΔHla
46_66
29_14
88_78
10_18
1_19
133_2
PB99
9_15
31_72
8_13
Hla: + - - + + - + + + - - -
CC8
CC5
(D)
MPO (pg/mL)
80000
60000
40000
20000
0
LPS-primed C57BL/6 BMNs
0.0167
Unstim
LPS
ATP
USA300
ΔHla
46_66
29_14
88_78
10_18
1_19
133_2
PB99
9_15
31_72
8_13
Hla: + - - + + - + + + - - -
CC8
CC5

Figure S4

Alpha-hemolysin Amino Acid Sequence Alignment

USA300 (AKR50780.1) - upper sequence Alpha-hemolysin CC5 - lower sequence

```
ADSDINIKTGTTDIGSNTTVKTGDLVTYDKENGMHKKVFYSFIDDKNHNKKLLVIRTKGT    60
ADSDINIKTGTTDIGSNTTVKTGDLVTYDKENGMHKKVFYSFIDDKNHNKKLLVIRTKGT    60

IAGQYRVYSEEGANKSGLAWPSAFKVQLQLPDNEVAQISDYYPRNSIDTKEYMSTLTYGF    120
IAGQYRVYSEEGANKSGLAWPSAFKVQLQLPDNEVAQISDYYPRNSIDTKEYMSTLTYGF    120

NGNVTGDDTGKIGGLIGANVSIGHTLKYVQPDFKTILESPTDKKVGWKVIFNNMVNQNWG    180
NGNVTGDDTGKIGGLIGANVSIGHTLKYVQPDFKTILESPTDKKVGWKVIFNNMVNQNWG    180

                           208
PYDRDSWNPVYGNQLFMKTRNGSMKAADNFLDPNKASSLLSSGFSPDFATVITMDRKASK    240
PYDRDSWNPVYGNQLFMKTRNGSMKAAENFLDPNKASSLLSSGFSPDFATVITMDRKASK    240

                                   275
QQTNIDVIYERVRDDYQLHWTSTNWKGTNTKDKWIDRSSERYKIDWEKEEMTN    293
QQTNIDVIYERVRDDYQLHWTSTNWKGTNTKDKWTDRSSERYKIDWEKEEMTN    293
```

Figure S5

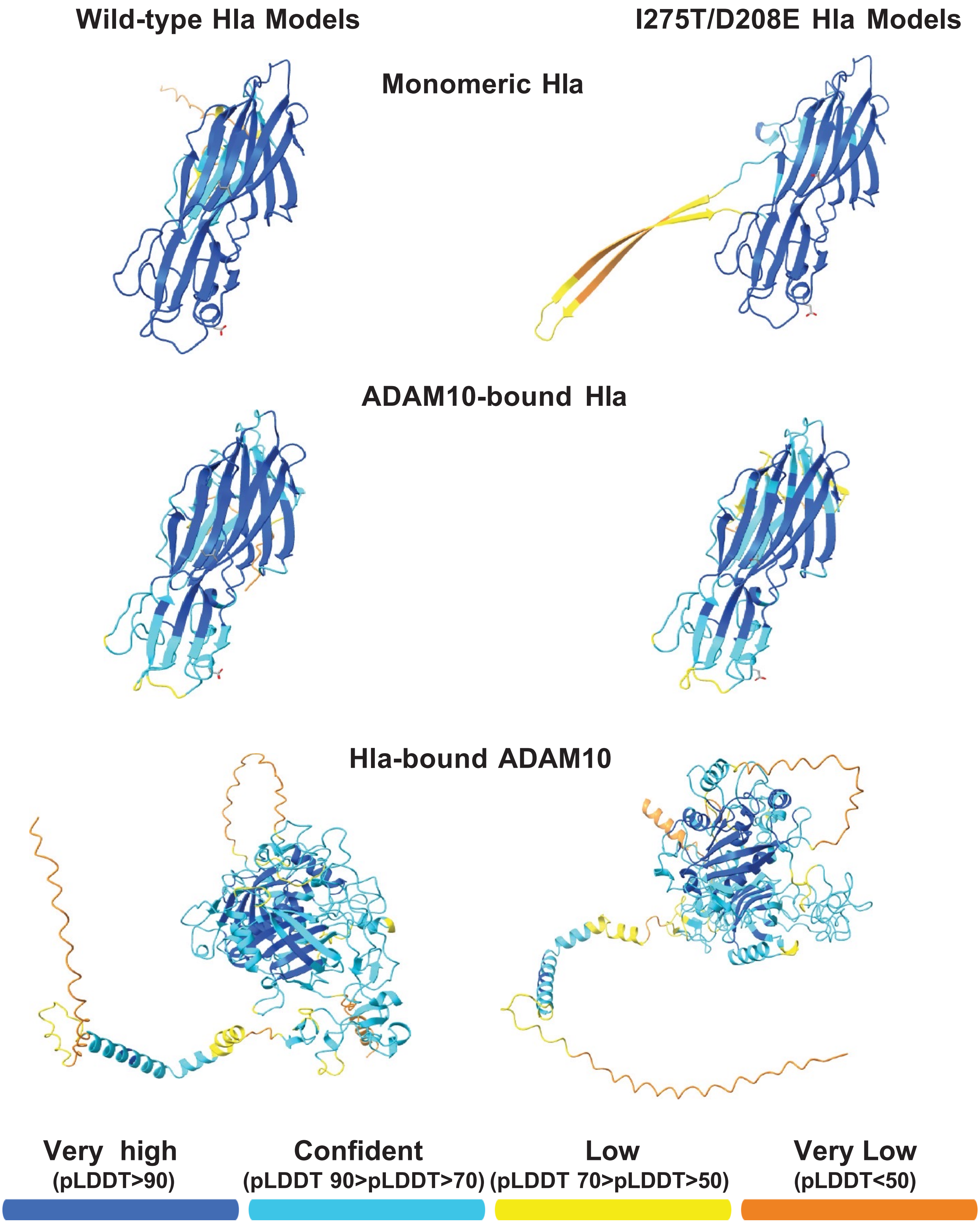
Wild-type Hla Models
I275T/D208E Hla Models
Monomeric Hla
ADAM10-bound Hla
Hla-bound ADAM10
Very high
(pLDDT>90)
Confident
(pLDDT 90>pLDDT>70)
Low
(pLDDT 70>pLDDT>50)
Very Low
(pLDDT<50)

Figure S6

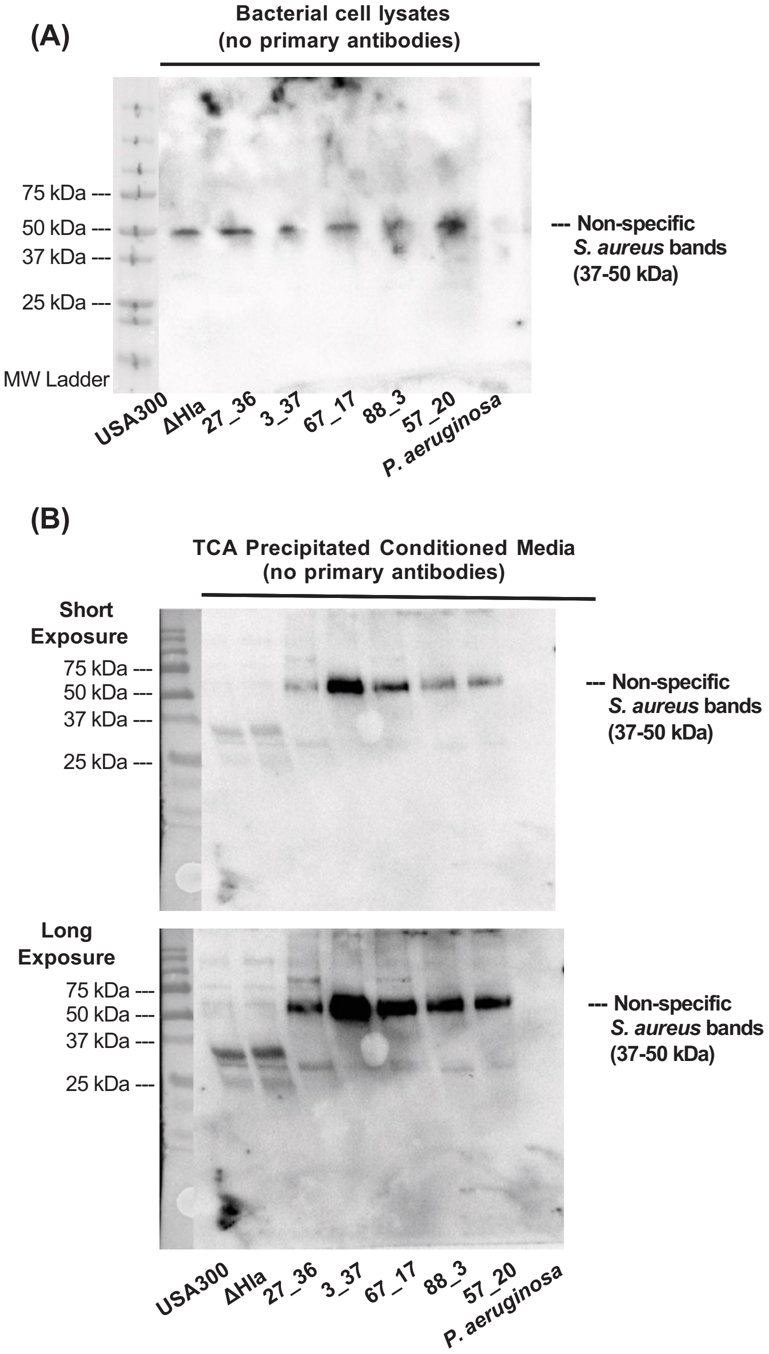
(A)
Bacterial cell lysates
(no primary antibodies)
75 kDa ---
50 kDa ---
37 kDa ---
25 kDa ---
MW Ladder
--- Non-specific
S. aureus bands
(37-50 kDa)
USA300
ΔHla
27_36
3_37
67_17
88_3
57_20
P. aeruginosa
(B)
TCA Precipitated Conditioned Media
(no primary antibodies)
Short Exposure
75 kDa ---
50 kDa ---
37 kDa ---
25 kDa ---
--- Non-specific
S. aureus bands
(37-50 kDa)
Long Exposure
75 kDa ---
50 kDa ---
37 kDa ---
25 kDa ---
--- Non-specific
S. aureus bands
(37-50 kDa)
USA300
ΔHla
27_36
3_37
67_17
88_3
57_20
P. aeruginosa

Table S1

Table S1: MRSA strains used in this study.							
Origins	Isolate ID	CC	ST	SCCmec cassette type	Characterisitics	Infection Demographic	
						Sex	Age
Massachusetts Eye and Ear (doi: 10.3389/fpubh.2020.00204)	46_66	CC8	ST8	IV	PVL+ ACME +	F	32
	29_14	CC8	ST8	IV	PVL+ ACME +	F	26
	88_78	CC8	ST3167	IV	PVL+ ACME +	M	28
	10_18	CC8	ST8	IV	PVL+ ACME +	F	47
	1_19	CC8	ST8	IV	PVL+ ACME +	F	39
	133_22	CC5	ST840	IV		F	86
	PB99	CC5	ST1176	IV		M	61
	9_15	CC5	ST5	IV		M	54
	31_72	CC5	ST5	II		F	87
	8_13	CC5	ST105	II		F	88
Nebraska collection (doi: 10.1128/mbio.00537-12)	MG2734	CC8			MRSA USA300 LAC parental strain		
	NE1354	CC8			MRSA USA300 a-hemolysin mutant		
